# Well-Come Back! Professional Basketball Players Perceptions of Psychosocial and Behavioral Factors Influencing a Return to Pre-injury Levels

**DOI:** 10.3389/fpsyg.2019.00222

**Published:** 2019-02-08

**Authors:** Cristiana Conti, Selenia di Fronso, Monica Pivetti, Claudio Robazza, Leslie Podlog, Maurizio Bertollo

**Affiliations:** ^1^BIND-Behavioral Imaging and Neural Dynamics Center, Department of Medicine and Aging Sciences, Università degli Studi G. d’Annunzio Chieti e Pescara, Chieti, Italy; ^2^Department of Psychological, Health and Territorial Sciences, Università degli Studi G. d’Annunzio Chieti e Pescara, Chieti, Italy; ^3^Department of Health, Kinesiology and Recreation, University of Utah, Salt Lake City, UT, United States; ^4^School of Science, Technology and Engineering, University of Suffolk, Ipswich, United Kingdom

**Keywords:** return to sport, return to performance, sport injury, injury rehabilitation, professional athlete

## Abstract

The psychological factors influencing a return to sport has gained increased research attention. In the current investigation, we explored professional basketball players’ perceptions of the psychosocial and behavioral factors facilitating a return to performance equal to or exceeding previous performance standards. We also sought to describe athletes’ experiences – both positive and negative – of returning to sport following injury recovery. Ten Italian professional male basketball players (age range 22–36 years), were retrospectively interviewed in relation to three time-periods: (1) from the commencement of rehabilitation to their first official competition, (2) the first official competition, and (3) the 6-months following the initial competition. Qualitative content analysis of the data revealed numerous themes across the three time periods. In regards to Period 1, participants indicated that social support, investment in rehabilitation and training programs, coping skills and motivation were fundamental in reaching pre-injury performance levels. During their first official game (i.e., Period 2), athletes reported that realistic performance expectations, focusing on the performance, positive emotions, motivation, arousal and social support facilitated their return to sport. Athletes, however, also described a predominance of factors that hindered their return to pre- injury levels (i.e., low confidence in personal abilities, decrements in skill execution and dysfunctional physical sensations). Moreover, participants typically described a substandard level of performance during their first competition back following injury. In recounting experiences during the 6 months following their first official game, basketballers reported improvements in skill execution and highlighted the importance of coping skills, motivation and social support. The process of restoring self-confidence in one’s ability to successfully perform was perceived as crucial in enabling participants to move beyond a mere return to sport to a return to high performance – that is, to reach a level of proficiency equal to or exceeding previous performance standards. Findings support the relevance of cognitive, emotional and behavioral responses highlighted in the Integrated Model and suggest the importance of addressing psychological factors throughout the return-to-sport process. Finally, results from the present study hold a number of practical implications for athletes’ aiming to achieve a return to pre-injury levels.

## Introduction

Return to sport following injury is a critical moment in the life of athletes and often represents the culmination of weeks to months (or longer) of rehabilitative efforts (Podlog and Eklund, 2006). For professional athletes in particular whose career prospects likely depend upon the ability to compete at or exceeding their pre-injury status, the culmination of injury rehabilitation is a return to full performance levels (Johnson and Podlog, 2014). Research on the return to sport has grown in recent years. Studies have highlighted the fact that returning to one’s pre-injury level is a complex and multifactorial process, directly and indirectly influenced by a range of physical, psychological and social factors (Podlog and Eklund, 2007; Ardern et al., 2016). Indeed, recent meta-analyses have demonstrated that athletes’ ability to attain or surpass pre-injury performance levels is not determined solely by the attainment of satisfactory clinical outcomes and physical functioning (Grassi et al., 2015; Ardern et al., 2016). Rather, these reviews have highlighted the relevance of psychological factors in predicting return to previous competitive levels, particularly in the context of elite sports where achieving optimal performance is of critical importance (Schilaty et al., 2016).

In an effort to examine the role of psychosocial factors in predicting recovery and return to sport outcomes, researchers have developed a number of conceptual models. One of the most prominent and well-tested models is the Integrated Model of Psychological Response to the Sport Injury and Rehabilitation Process (Wiese-Bjornstal et al., 1998). The model which was developed based on adaptations of stress and coping theory (Lazarus and Folkman, 1984) proposed that the way an athlete appraises their injury (cognitive appraisal) determines subsequent emotional (e.g., frustration, denial, anger, happiness, relief) and behavioral responses (e.g., adherence to rehabilitation, use of psychological skills). Further, a host of personal (i.e., injury specific, individual difference, demographic, physical) and situational (i.e., sport, social, environmental) factors are proposed to influence cognitive, emotional and behavioral responses to injury. The post-injury psychological response process is considered cyclical and dynamic, reflecting the recursive influence between thoughts, feelings and actions. Finally, Wiese-Bjornstal et al. (1998) proposed that psychological responses influence short and long-term psychosocial and physical recovery outcomes. As indicated, a key recovery outcome, particularly for elite performers, is the ability to return to or surpass pre-injury performance levels.

Strong empirical support has emerged for numerous contentions articulated in the Integrated Model. Specifically, scholars have found a multitude of injury-related cognitions, emotions, and behaviors from initial injury occurrence to a return to play; links between personal and situational factors and athlete responses to injury (for a review see Brewer, 2007); and relationships between responses to injury and rehabilitation outcomes (for a review see Brewer, 2010). With regard to cognitive appraisals and emotional responses, consistent findings suggest a range of negative injury appraisals (difficulties managing pain and loss of physical function, social isolation, summoning motivation) and emotions (devastation, frustration, helplessness, fear, depression, and resentment), with positive perceptions typically increasing as recovery progressions become apparent (Carson and Polman, 2008; Stoltenburg et al., 2011; Ruddock-Hudson et al., 2012, 2014). Of particular relevance to the current study, increasing evidence supports links between psychosocial and behavioral factors and recovery outcomes (e.g., Ardern et al., 2011, 2013a). For instance, researchers have shown that factors such as motivation (Podlog and Eklund, 2005), re-injury apprehensions (Ardern et al., 2011, 2012, 2013b), and psychological readiness (Ardern et al., 2014) predict return versus non-return as well as the quality of athletes’ post-injury performances.

Although research on the recovery outcomes portion of the Integrated Model is growing, the majority of research in this area has been quantitative with comparatively few qualitative investigations (Podlog and Eklund, 2006; Carson and Polman, 2012; Tjong et al., 2013; Podlog et al., 2015). Given that athletes are ultimately the ones making a return to competitive activity, eliciting their perceptions and beliefs about the factors that facilitate their return to pre injury levels is of clear relevance. Moreover, of the studies examining psychosocial factors influencing return to competitive sport levels, few have focused on professional athletes, and to our awareness, none have focused on the sport of basketball. As Heil (1993) asserts, elite athletes can serve as templates for others given their strong achievement orientation, their task focus, and ability to handle pain. With respect to a focus on basketball players, the dearth of research on athletes in this sport is rather surprising considering it is one of the most popular sports in the world and the number of young participants is rising (Harmer, 2005); it is characterized by high levels of physical contact; and there appear to be increases in the prevalence of injuries over time (Trojian et al., 2013).

Given the limitations and gaps of previous research, as well as the comparatively small amount of work focused on the recovery outcomes portion of the Integrated Model (Wiese-Bjornstal et al., 1998), our aim in the present investigation was twofold. First, we wanted to ascertain professional basketballers’ perceptions of the psychosocial and behavioral factors they perceive as critically important in reaching or surpassing pre-injury performance levels. Second, we sought to describe basketballers experiences of a return to sport – including positive and negative experiences – by examining three time periods, namely, from the commencement of participants’ rehabilitation to their first official competition (Period 1), the first official competition (Period 2), and the time frame covering the 6-months following the first official competition (Period 3). Although the three distinct periods examined in this investigation, are in nature more continuous than discrete, we chose to examine the emergence of specific psychosocial and behavioral factors in each period of the return to sport experience. This decision was based on our interest in understanding athlete perceptions of the precursors (i.e., antecedents) contributing to a return to previous or higher performance levels.

## Materials and Methods

### Participants

Participants were selected based on the following inclusion criteria: (1) male gender; (2) a basketball player in the Division I (A1) professional level of the Italian Basketball League; (3) incurred a moderate-severe sport injury requiring a minimum of 2-months absence from practice; (4) returned to sport following injury for at least 6-months; (5) had not experienced re-injury in the 6-months following the return to the first official game; (6) returned to perform at level equal to or surpassing pre-injury abilities. The final criterion was determined based on whether players’ had resumed participation with a professional club in the highest Italian Basketball League and performance statistics taken during matches (e.g., minutes game, points, field goals attempted, field goals made, rebounds, assists, shots on net, plus/minus statistic). Moreover, since gender is listed as a personal factor influencing responses to injury and the rehabilitation process in the Integrated Model (Wiese-Bjornstal et al., 1998), we chose to focus exclusively on male athletes to obtain a homogeneous sample. Such criteria resulted in the recruitment of ten Italian male professional basketball players, from different teams in the first division (A1) of the Italian Championship Basketball Federation. As highlighted in Table 1, players ranged in age from 22 to 35 (*M* = 27.7; *SD* = 4.49), spent at least 3 h of training per day and competed in one or two matches per week. Participants had suffered a sport injury resulting in a range of 2–9 months absence from training or competition (see Table 1). Seven athletes experienced traumatic injury (e.g., rupture of anterior cruciate ligament), while three athletes were affected by degenerative diseases (e.g., knee cartilage and spinal disk herniation).

**Table 1:** Participant characteristics.

Athlete	Age	Hours of training (per day)	Matches (for week)	Injury sustained	Nature of injury	Absence from practice (months)
1	23	3	1	FAI, Femoro acetabular Impingement	degenerative	6
2	35	4	2	Cervical Disk Herniation	degenerative	5
3	31	3/4	1	ACL Rupture [sx]	traumatic	6
4	30	3/4	2	ACL Rupture [sx] Torn Meniscus [sx]	traumatic	7
5	33	3	1	ACL Rupture [sx] Torn Meniscus [sx]	traumatic	8
6	30	4	2	Knee Cartilage Transplant [dx]	degenerative	5
7	23	3	1	Adductor Strain [sx]	traumatic	2
8	27	4	2	ACL Rupture [dx]	traumatic	7
9	22	3	1	ACL Rupture [dx]	traumatic	9
10	23	3	1	Quadriceps Strain [dx]	traumatic	3


### Interview Guide

A semi-structured interview guide was developed based on the psychology of sport injury literature (Podlog and Eklund, 2006; Wadey and Evans, 2011; Carson and Polman, 2012; Podlog et al., 2013) and the first author’s previous experience consulting with injured athletes. The interview included demographic questions as well as those pertaining to the three time-periods described above (see Appendix). Athletes were asked to focus on the psychosocial and behavioral factors facilitating their return to pre-injury levels during each time-period of interest. When necessary, further probe questions (e.g., What do you mean by...? Could you please explain that in more detail?) were used to clarify points raised in the interviews and to ensure consistent responses in terms of depth and complexity (Patton, 2002).

### Procedures

Participants were recruited by phone, email, or in person using the informal and professional network of the authors. At the time of data collection, the first author worked as a consultant with a professional basketball club, which enabled her to contact numerous injured professional athletes, health professionals, and rehabilitation centers. All participants were provided with an information sheet detailing the study purposes and procedures, informed of the reason for recording the interviews, and assured complete confidentiality and anonymity. All athletes who were contacted agreed to participate. Following receipt of written informed consent, participants were involved in a semi-structured individual interviews conducted via skype with only the athlete and first author present. All interviews were tape-recorded and lasted between 40 and 59 min (*M* = 48 min). Numerical codes were used to ensure the anonymity of athletes. At the end of the session, athletes were debriefed and thanked for their participation. This study was carried out in accordance with the recommendations of the Institutional review board (IRB), and all subjects gave written informed consent in accordance with the Declaration of Helsinki. The study was approved by the ethical committee for biomedical research of Chieti-Pescara University (ref. n. 10-21/05/2015).

### Data Analysis

All interview data were subjected to qualitative content-analysis following the procedure outlined by Thomas (2006), and used by Bertollo et al. (2009), integrating the suggestions provided by Flick (2018). Previous literature on the topic (Carson and Polman, 2012) and the structural framework derived from the three time-periods of interest were used as a guide to code the data. Specifically, the following six-step process was conducted. First, audio-taped interviews were transcribed verbatim, which resulted in 107 pages of single-spaced text. Next, the interviews were read and re-read by the first and last authors to obtain familiarity with them. Third, the researchers independently, extracted raw data themes (significant statements and phrases that directly related to psychosocial and behavioral factors) for each period considered (i.e., from rehabilitation to first official competition, first official competition, the following 6th months). The interview material was content-analyzed using the Microsoft Excel program (Dey, 1993). An athletes verbatim statement that referred to a single coherent idea was considered a text unit. Each text unit was coded according to its underlining meaning into raw data themes. Fourth, the researchers individually coded and grouped the raw data themes into meaningful higher-order themes and general dimensions, on the basis of their underlining conceptual similarities. The choice of categories themes/dimensions followed either a top-down, deductive strategy, with some themes emerging from the literature (e.g. Santi and Pietrantoni, 2013), or a bottom-up, inductive approach, with some themes emerging from the data, following repeated reading of the interview transcripts. One text unit could belong to various themes/dimensions, whereas other text units that did not appear relevant to our primary questions of interest were not coded at all. In the penultimate stage, the researchers met and compared their coding schemes, discussing their rationale in classifying particular text units within specific themes as well as the appropriateness of the theme labels. Finally, inconsistencies and disagreements between the two authors coding the data were resolved through discussion with a third investigator (MP), experienced in qualitative content analysis. Moreover, to enhance the trustworthiness of our qualitative data analysis, the third author reviewed the analyses, questioned the logic of categorization decisions, prompted discussions, and explored alternative explanations (Miles et al., 2014).

## Results

Results are divided into three sections corresponding to our time-periods of interest, namely from the commencement of rehabilitation to participants first official competition (Period 1), the first official competition (Period 2), and the 6 months following the first official match (Period 3).

### Period 1: Perceptions of the Factors Influencing a Return to Competition

**FIGURE 1:**
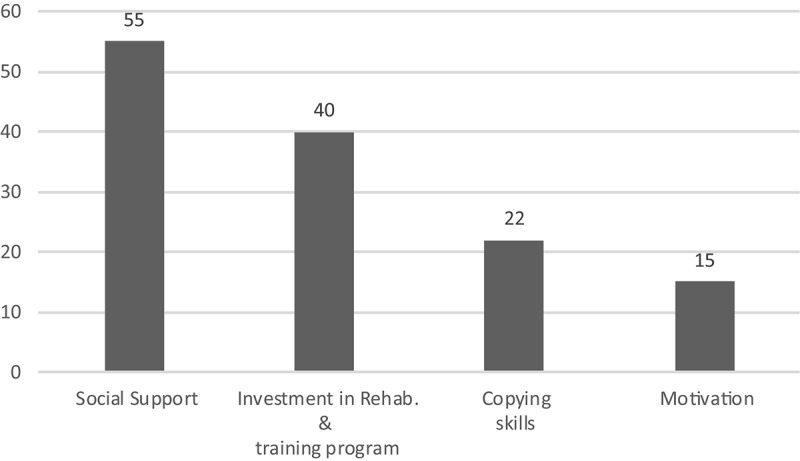
Number of raw data themes for each general dimension during Period 1.

Analysis of the data revealed 133 raw data themes pertaining to athlete perceptions of the psychosocial and behavioral factors influencing their ability to regain or surpass pre-injury levels. In thinking about such factors, the interviewer asked participants to contemplate the time-period ranging from the commencement of rehabilitation until the return to competition, that is, until participants’ first official game. Raw data themes were classified into seven higher order themes grouped into four general dimensions, namely: (1) social support; (2) investment in rehabilitation and training programs; (3) coping skills; and (4) motivation. A list of example raw data themes, higher-order themes, and general dimensions is provided in Table 2. Moreover, Figure 1 reports the number of raw data themes for each general dimension during Period 1.

**Table 2:** Raw data themes, higher order themes and general dimensions related to Period 1 (from the commencement of rehabilitation to first official competition).

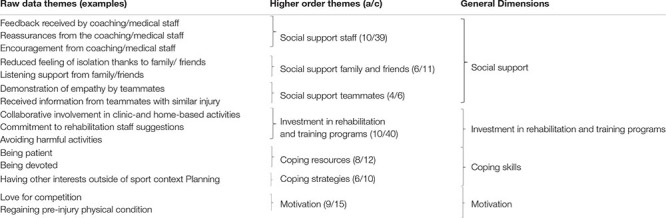

*Note:a = number of athletes citing a raw data theme; c = number of raw data themes.*

#### Social Support

Athletes described the pivotal role of a supportive network of team staff (coaches, athletic trainers and sport medicine professionals), teammates, family and friends in facilitating their return to pre-injury levels. Such individuals were suggested to be instrumental in providing various types of social support, for example, informational (e.g., giving advice, feedback, suggestions, and information to increase athlete’s awareness of the return process and reduce concerns), emotional (e.g., listening, reassuring, and demonstrating empathy) and motivational support (e.g., encouraging athletes to overcome setbacks, recognizing the missed contribution of the injured athletes). All athletes emphasized the crucial role of physiotherapists and athletic trainers in providing informational, emotional, and motivational support. An interesting finding was that participants commonly reiterated that such specialists took time to inform them of and elucidate potential return to competition scenarios during the rehabilitation period (e.g., the time needed for the body to be at the same functional capacity as pre-injury). For instance, athlete 1 highlighted “If I think to my come back, I need to recognize that the physiotherapist was very helpful. He pushed me to create a goal and to be more aware about my condition, actual and future.” Similarly, athlete 6 remarked, “I remember a few weeks before my first competition, my athletic coach had spent some time giving me information on the possible physical conditions of an athlete who comes from a long time injury and comes back to compete; it was very useful for me to create ideas and expectations about what I would have found in the court.”

The emotional and motivational support received by family, partners, and friends was valuable in mitigating feelings of isolation as described by athlete 4, (“Having around people who support you like family and friends, is really comforting and helps you to feel not alone”) and athlete 5 (“I felt a total support from my family to continue my rehab, they strongly believed in me and in my efforts”). Teammates were also a source of motivational support. Epitomizing the belief of others, athlete 10 stated he felt extensive support from teammates because they were looking forward to having him back on the court. Participants also reported that teammates who had experienced similar injuries were valuable sources of emotional and informational support. As athlete 3 indicated, “I remember that one of my teammates was texting me all the time. He had a similar injury before: those people who have had the same experience in the past can really understand you and give you important information about the return process.”

#### Investment in Rehabilitation and Training Programs

Participants described the importance of being dedicated to their rehabilitation and subsequent training by actively participating in rehabilitation and training sessions, completing clinic-and home-based activities, listening to rehabilitation staff suggestions and avoiding potentially harmful activities. For instance, athlete 2 suggested that “working hard, following the recommendations from healthcare providers and avoiding the tendency of be overly adherent to a rehab regime, are fundamental to feel that your body is ready again to compete”. Similarly, athlete 9 commented that *“*being dedicated, not taking anything for granted, not doing less but exactly what practitioners have suggested to you, even when you are at home; this is the best way to have a successful return”. All participants believed that their diligent investment in rehabilitation and training programs was fundamental in achieving functional body perceptions (i.e., high physical performance capabilities), increasing confidence in the injured body part, and promoting personal involvement in planning their return.

#### Coping Skills

Basketballers indicated that personal coping resources (i.e., intra-individual characteristics) and well-developed coping strategies (i.e., intentional tactics employed to address injury stressors) during the rehabilitation were key factors enabling a return to pre-injury levels. Coping skills were identified as crucial to effectively complete rehabilitation and training programs, to attenuate stressful situations, and to regulate emotions associated with the typical “ups and downs” of the rehabilitation period. Athletes emphasized the salience of particular coping resources or trait-like characteristics, such as optimism, patience, determination and devotion. For instance, athlete 8 commented, “If you are the kind of person with an optimistic outlook, as I see myself, it helps you experience greater focus and less concern about the rehabilitation process.” Moreover, participants reported, active acceptance, rational thinking, problem-solving, having other interests, planning and setting effective goals as useful active coping strategies. As athlete 5 remarked, “In my opinion it is important and useful to have a life outside of basketball. Having other interests, hobbies, and friends helped me to not be focused on the injury, especially when I was feeling down.”

#### Motivation

Participants considered motivation an essential ingredient in regaining physical performance standards and returning to pre-injury levels. In particular, all participants believed that their love of the competition and the motivation to regain previous physical conditions helped energize their return to sport and pushed them to give “100% effort” in recovering from their injury. Motivation was also deemed to be fundamental in managing the challenges, setbacks and difficulties associated with the rehabilitation time-frame. The following statements epitomized this idea: “I was motivated to train hard, to come back as soon as possible at a high standard of physical conditioning” (athlete 7), and “my motivation had pushed me to keep going, especially when I felt the boredom of the rehabilitation exercises or the difficulty of the training program” (athlete 6).

### Period 2: Athlete’s Experience in Returning During the First Official Competition

**FIGURE 2:**
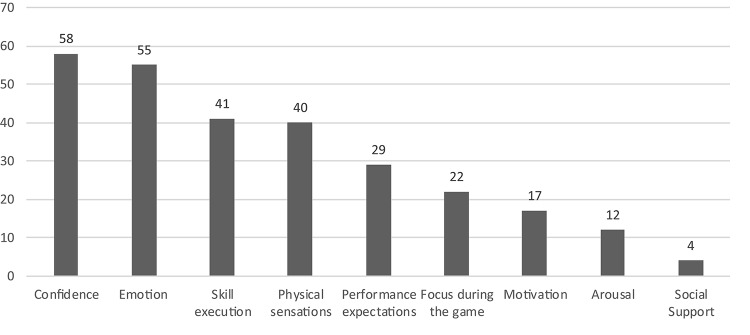
Number of raw data themes for each general dimension during Period 2.

Analysis of the transcripts related to the first official game highlighted 288 raw data themes which were grouped into 17 higher order themes, and nine general dimensions. The latter included: (1) confidence; (2) emotions; (3) skill execution; (4) physical sensations; (5) focus during the game; (6) performance expectations; (7) arousal; (8) motivation; and (9) social support. A list of example raw data themes, higher order themes, and general dimensions is provided in Table 3. General dimensions pertaining to motivation and social support, highlighted in Period 1 also emerged in relation to Period 2. Although athletes conveyed slightly different points of emphasis in relation to motivation and social support across the two time-periods (see raw data themes in Tables 2, 3), given space limitations, we only address the six novel general dimensions described in regards to Period 2. Moreover, Figure 2 reports the number of raw data themes for each general dimension during Period 2.

**Table 3:** Raw data themes, higher order themes and general dimensions related to Period 2 (first official competition).

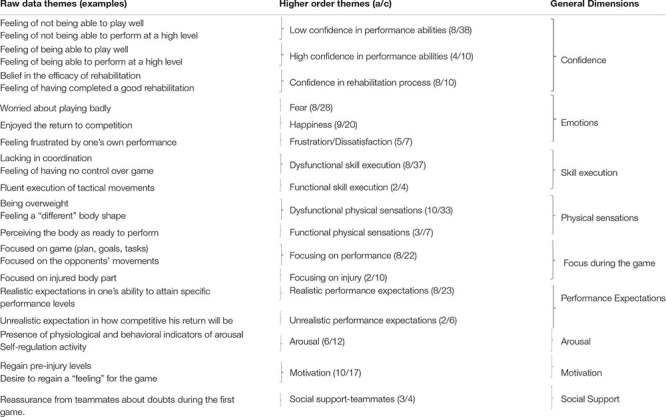

*Note:a = number of athletes citing a raw data theme; c = number of raw data themes.*

#### Confidence

In reflecting on their experiences during their first official league match, athletes articulated the importance of two confidence dimensions, namely confidence in the effectiveness of their practitioner/rehabilitation program, and confidence in their personal performance capabilities. Virtually all participants indicated that a key reason why they felt confident upon their initial return to competition was because they believed in the abilities of their rehabilitation practitioners and in the effectiveness of the work completed during their rehabilitation programs. Comments by athletes 8 and 1 nicely captured the confidence participants felt in their rehabilitation practitioners and programs: “I was certain of the work of the physiotherapists and the trainers, satisfied of the physical outcome of rehabilitation” (athlete 8), and “the months before my first competition I worked hard with very strong expert professionals, I believed in them, totally, and in our work… it was a positive thought when I was on the bench ready for my first match after the injury” (athlete 1).

Despite the fact that participants expressed confidence in the abilities of their practitioners and the effectiveness of their recovery regimens, they nonetheless indicated a lack of belief in their performance capabilities and in their ability to execute skills. For instance, athlete 10 commented: “my body was ok, ready...but I felt I was not able to do what I was usually do. Definitely, it was my first feeling; not being able to do the things that I used to do was the most hurtful experience”. Echoing this sentiment, athlete 3 remarked, “I played and I realized that I was not as good as I thought I should be; my confidence was very low and I remember the doubts I had during the first game on my ability to play.”

#### Emotions

The inability to execute tasks one had previously performed with relative ease, typically resulted in feelings of frustration and dissatisfaction. As athlete 5 articulated, “sometimes I got frustrated because I was trying to do the things I used to do before [the injury], but I wasn’t able to.” Somewhat surprisingly, the intensity of athletes’ negative affect was not extremely high. Interview comments revealed that some rehabilitation specialists had prepared returning basketballers for the fact that their performance might not be as high as pre-injury levels upon their immediate return. Epitomizing this sentiment, athlete 3 commented: “of course it was frustrating the feeling of not being able to do what you want and should be able to do, but I had been prepared by the professionals on this possibility.”

A range of other negative emotions were expressed in relation to participants’ return to competitive play. In particular fear and concerns regarding one’s ability to attain or surpass preinjury performance levels, to execute sport-specific skills, and to appear fit and decisive in one’s on-court decision making were repeatedly mentioned. Speaking to his concern about reaching or surpassing pre-injury levels, athlete 7 suggested: “there was fear too. In setting your foot again on the...surely there was a bit of fear, fear about not being the athlete I was before [the injury]”. Interestingly, the fear of sustaining a new injury was only identified by two participants, perhaps an indication of the quality of the work of treatment team members.

Despite a predominance of negative emotions, participants also reported feelings of happiness and “joy [associated with] being back on the court” (athlete 4) and suggested that positive emotions were a “natural reaction for once again doing something that you love [to do] and have waited for a long time” (athlete 1). Characterizing the thoughts of others, athlete 2 stated: “I was happy because I wanted to return to play and I could play again…you know, when you don’t step on the court for a long time, you are looking forward to going back on it.”

#### Skill Execution

Participants consistently reported decrements in skill execution during their first game back. For instance, athletes highlighted feeling “more slow and clumsy,” “less reactive,” lacking in “coordination,” and making “bad shot selections.” As athlete 6 commented: “I’ve been feeling that I had bad shooting selection; some movements which I used to be familiar with, became strange to me.” Athlete 9 also remarked: “The positions on the court, the angles…you saw things, you did things with a millisecond delay, then this [the delay] makes the difference.” Athletes emphasized that their perception of diminished skill execution was heightened when they compared their performance with those of their teammates, especially, in instances when participants made their first competitive appearance in the middle of the season against athletes with substantially more match play. Although almost all athletes highlighted decreases in skill execution during the first official game, two indicated an ability to effectively execute game-specific skills. These two athletes, did, however, suggest that their initial assessment of their skills may have been overinflated due to the euphoria of being able to resume their competitive play. Athlete 5, nicely described the point: “Honestly, at the end of the first game I had very positive perceptions of my performance: I thought I had already returned to my pre-injury level. The experience of the following games, however, was very different and I felt the quality of my skill execution was much lower. I think it [my initial perception] was because when I played [in the first game] I was guided by positive emotions and fan support. It was only during the second and third games, that I realized that my true level of performance was not what I experienced during my first game.”

#### Physical Sensations

Despite attaining physical standards and clinical levels required for medical clearance to return to competition, almost all athletes indicated that their body was not completely ready to perform at a high level and reported dysfunctional physical sensations. According to athlete 3: “I felt my lungs burning because I was not used to running for a long time and I was a bit overweight.” While some participants acknowledged that indications of their physical limitations were instructive in identifying current performance levels and the need to avoid rushing the return, almost all athletes expressed discomfort with these perceptions. Moreover, some participants expressed a strange feeling linked to having a different body shape (often bigger muscles) and did not recognize their body immediately. The comments of athlete 1 nicely captured this body perception: “I was very thin before the injury; when I came back I had more muscles because I had worked a lot in the gym and so I was like a different player; I didn’t have the same speed and explosiveness. I had gained, of course, in mass, but at the beginning it was a bit hard to accept it, because I didn’t recognize my body.”

#### Focus During the Game

Athlete interviews also revealed issues surrounding attentional focus during the first official game. All participants recognized that effective performance required “… the athlete to pay attention to the right things at the right time” (athlete 5). The majority of participants reported a focus on performance related cues and situations. Indicative of the comments of others, athlete 2 stated: *“*when the game started I didn’t think about injury, just about the ball and about doing the best I could. I am a professional athlete and this was my instinctive behavior.” Other participants reiterated a similar suggestion, commenting that they felt totally focused on the essential elements of the task to be performed (athlete 4) and on what they had to do (athlete 6). Interestingly, many of the basketballers shared the experience of the difference between the practice and their first game, emphasizing that “even though during training the focus was often on the injured body, especially to monitoring the physical condition [of the injury], during the game the attention was totally on the performance” (athlete 1).

However, two athletes indicated an internal focus on their injured body part; for instance, athlete 9, who sustained an ACL rupture, indicated that he was not focused on performance and his focus was primarily on sensations related to his formerly injured knee joint. The inward focus on injury during the game was deemed dysfunctional by the two athletes citing this concern. In particular, both athletes suggested that their inward focus on the injury sites reduced their capacity to attend to important task-relevant cues, game plans, and their performance goals. Indeed, athlete 10 commented that his attention on the injured part was detrimental because he could not concentrate on what he planned to do to during the match, while athlete 9 remarked “that focus [on the injury] during the match reduced my ability to respond quickly to what was happening around me in the court.”

#### Performance Expectations

Participants discussed the importance of realistic performance expectations (e.g., attaining specific goals or performance levels) during the first game back. Several athletes believed they possessed realistic performance expectations, which they attributed to the information they received from health care providers and to previous experiences returning to sport after injury. Such information and past injury experiences, helped athletes realize that a return to pre-injury levels would likely take some time. For instance, athlete 7 commented that the transition from rehabilitation to competitive performance did not happen immediately, but was more of a gradual progression. In support of this contention, Athlete 6 stated: “I knew that during my first game my movement would have been slower, less fluid, than under normal conditions. You have to be honest with yourself about your initial performance after injury.” Despite frustrations over a disconnect between athletes’ actual and desired initial performance, participants still believed that their first game back was important in providing a test or check of their actual readiness levels to resume competitive basketball. The comments of athlete 10 typified this idea: “only when an athlete is going to play his first official game, can he really check his state of psychological readiness.”

#### Arousal

Two important aspects related to the issue of arousal were revealed in participant interviews, namely, an awareness of one’s arousal levels and the importance of self-regulating one’s arousal levels. A number of players described a vivid awareness of various physiologic (e.g., “sweaty palms and a shortness of breath,” athlete 5; “an increase in heart rate,” athlete 9; butterflies” in the stomach, athlete 10), and behavioral indicators of elevated arousal (e.g., “movements excessively energetic,” athlete 3; “arms and neck shaken repeatedly,” athlete 7) related to their first game. Some participants interpreted their high arousal levels as facilitative, for example, athlete 8 who indicated “My body was full of adrenaline, I was completely energized and ready to play.” Conversely, other athletes perceived their elevated arousal states as unpleasant and debilitative to their performance such as athlete 5: “I was overly agitated and I realized that my performance could be compromised by my over-aroused body. I urgently felt the need to doing something to change or reduce this unfavorable state.”

The ability to down-regulate one’s arousal was another salient issue reported in athlete interviews. Basketballers indicated use of techniques such as deep breathing and cognitive techniques (i.e., self-talk) as nicely captured by athlete 10: “When I recognized that the excessive activation of my body could have had a negative impact on performance, I used slow, deep breathing. Just for a minute, but it was very useful to get back into the flow of the game.” Along these lines, athlete 7 stated, “My body activation was not ok. To reduce the high levels of excitement I was experiencing, I tried to use positive self-talk to replace my attention on the right path.”

### Period 3: Athlete’s Experiences in Returning to Sport During the Six Months Following the First Official Competition

**FIGURE 3:**
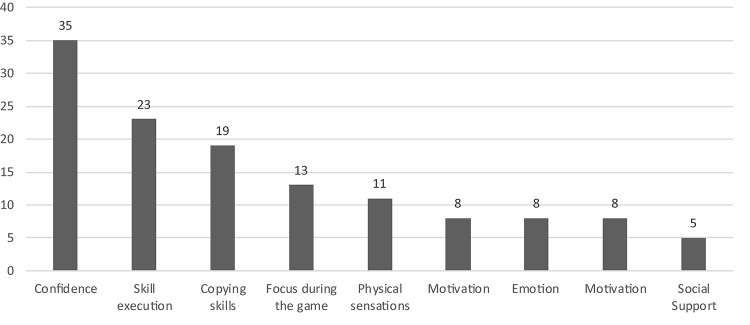
Number of raw data themes for each general dimension during Period 3.

Content analysis of the interviews related to the period following the initial 6 months of the return to competition yielded 124 raw data themes which were grouped into 15 higher order themes, and eight general dimensions: (1) confidence, (2) skill execution; (3) coping skills; (4) physical sensations; (5) focus during the game; (6) motivation; (7) emotions; and (8) social support. A list of example raw data themes, higher-order themes, and general dimensions is provided in Table 4. Once again, similar general dimensions emerged across Periods 2 and 3, albeit with different points of emphasis. Given the salience of confidence, skill execution, coping skills and physical sensations, during Period 3, we provide further elaboration of these dimensions. In regards to the issue of athletes’ game-related focus, all participants reported a functional focus on game-related cues as well as opponents’ movements. No mention of an internal injury focus was described in reference to Period 3. With respect to motivation, as was the case in Period 2, participants reiterated a continued interest in regaining pre-injury performance standards and proving one’s performance abilities. Finally, social support from coaching/medical staff and teammates in the form of feedback, encouragement and inspiration were all deemed instrumental in overcoming return-to-sport challenges in the initial 6-month period. Moreover, Figure 3 reports the number of raw data themes for each general dimension during Period 3.

**Table 4:** Raw data themes, higher and general dimensions related to Period 3 (from the first official game to 6 months post-return).

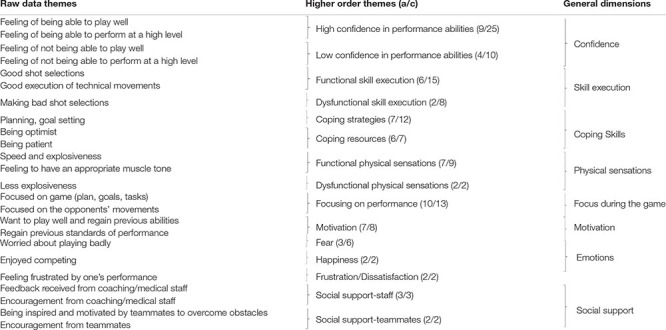

*Note:a = number of athletes citing a raw data theme; c = number of raw data themes.*

#### Confidence

A key finding to emerge during Period 3 was an increase in athletes’ confidence in their performance capabilities. As athlete 6 commented, “going on with the months, continuing to work, I found the ‘fluidity’ of everything and gradually I gained confidence in my ability to play at a high level.” Similarly, athlete 9 remarked “match after match, I felt that I came back to believe in my skills and think: yes, I can do it, I can perform again at a high level.” Participants attributed their renewed sense of confidence to the experience of successful on-court performances and to improvements in technical and mental aspects of their game. Reinforcing the significance of positive performance experiences, athlete 8 suggested, “I needed about 4 months to get 100% on the court because my game is mainly determined by physical abilities like speed, characteristics that I had not recovered before. These physical abilities gradually returned and I believed my performance was of a higher quality; consequently [I had] greater confidence in my abilities.” In regards to the importance of technical/mental improvements, athlete 3 indicated, “the obstacles I encountered during the months after the first game in order to reach a high standard, had some advantages. For example, I realized that I had to be more focused on the game than I was before the injury and, absolutely, psychologically stronger. It [the injury] increased my mental toughness, improved my technical awareness and helped me become more confident in my abilities.” Athlete 1 underscored a similar point in suggesting “when I came back I had more muscles and I had to change my way of performing a little bit; at the beginning it was not easy, but gradually this [tactical adaptation techniques] made me feel I had more “weapons” and increased my level of confidence”. While some participants acknowledged a low level of confidence in personal abilities during period, such experiences were restricted to the second and third games back following injury.

#### Skill Execution

According to the basketball players, increases in skill execution generally occurred with each passing game and were accredited to the recovery of particular movement patterns, technical/tactical abilities, or being “in-sync” with fellow teammates. As athletes gained increased competitive opportunities, they reported “feelings of being one with the court” (athlete 3) or “feeling good timing” (athlete 7). Athlete 10 emphasized the value of gradual progressions in regaining effective skill execution: “I perceived the fluent execution of tactical skills after 3 or 4 games. It was a gradual process.” While participants consistently articulated improvements in skill execution following the first 6 months after their return, two athletes reported poor skill execution, specifically in relation to the challenges associated with shot selection during the game.

#### Coping Skills

Considering the multitude of challenges and extended time-frame associated with a return to pre-injury levels, it was not entirely surprising that participants emphasized the importance of various coping skills. In particular, participants suggested that personal coping resources such as “being patient and maintaining a positive attitude” (athletes 6) were invaluable in maintaining motivation and positive emotions as they attempted to overcome gaps between current and desired performance levels. Likewise, use of intentional coping strategies such as a rational/positive thinking, and goal-setting were deemed essential in overcoming return-to-sport challenges and demands. Indicative of others, athlete 2 commented that “setting appropriate goals with the help of the coaching staff, was essential to continue working and gradually improving skills and to achieve high-quality performances.” Moreover, participants suggested that particular skills and attitudes cultivated over the course of their professional careers (e.g., a high-level of body awareness, a positive attitude toward training, taking personal responsibility for one’s own preparation), were relevant for being an active subject in the process of return. Participants also indicated that paying attention to significant factors during the warm-up and the game enabled them to achieve high levels of post-injury performance.

#### Physical Sensations

Interestingly, athletes reported decreases in dysfunctional physical sensations over the course of their first 6-months back following the return to competition. In particular, virtually all participants reported some form of adaptive physical sensations/abilities such as a greater ability to “physically perform” (athlete 6), “having appropriate muscle tone” (athlete 1), “being more explosive” (athlete 7) and, “having good sensations from the body” (athlete 3).

## Discussion

The purpose of the current study was to explore athlete perceptions of the psychosocial and behavioral factors facilitating (or hindering) a return to pre-injury performance levels. The study makes important contributions to the literature in several respects. To the best of our knowledge, this was the first investigation analyzing the experience of returning to performance using a specialized sample of professional athletes, individuals who were successfully able to achieve or surpass their pre-injury levels. Second, this investigation provides relevant insights into the particular psychological factors which top-level athletes deem essential in achieving a return to performance equal to or exceeding previous performance standards. Third, the consideration of three time-periods allowed us to examine the psychosocial factors that athletes believe facilitated (or hindered) the return to performance (i.e., the same or higher pre injury level). Fourth, our retrospective approach enabled us to ascertain athlete views among those who experienced the “ups and downs” of a return to performance and who had the benefit of “perspective” in thinking about their experiences. Fifth and finally, from a practical standpoint, understanding the relationship between psychosocial and behavioral factors and return to performance could assist injured athletes and practitioners in addressing critical factors during rehabilitation that maximize the likelihood of achieving or surpassing pre-injury levels.

A novel aspect of the study was that such experiences were examined in relation to specific time points/periods following the completion of athletes’ formal rehabilitation. An elite sample of professional Italian basketball players who had successfully returned to and/or surpassed pre-injury levels provided original insights into our questions of interest. Consistent with assumptions from the Integrated Model (Wiese-Bjornstal et al., 1998), athletes emphasized the importance of situational (i.e., social support) and personal (i.e., investment in rehabilitation and training programs, coping skills and motivation) factors in describing their perceptions of a return to competition (i.e., Period 1). Specifically, athletes recognized the importance of emotional and motivational support received from family, friends, and teammates, especially those who had sustained a similar injury. Of particular relevance to basketballers was the vital support provided physiotherapists and athletic trainers. This finding echoes past research in which athletes indicated greater satisfaction with and support received from these practitioners in comparison with coaches or fellow athletes (e.g., Clement and Shannon, 2011; Fernandes et al., 2014). In particular, the current study suggests that support from qualified professionals could be instructive in providing information and education about return-to-competition processes, which could in turn, promote greater perceptions of control and knowledge about what to anticipate upon one’s return to sport. Further research examining the benefits of informational support for athletes transitioning back into the competitive arena is warranted.

Participants also reinforced the necessity of adhering to prescribed rehabilitation exercises and guidelines, a finding reinforcing previous research demonstrating the importance of rehabilitation in facilitating psychosocial/physical rehabilitation outcomes and a greater likelihood of return to pre-injury levels (Wiese-Bjornstal et al., 1998; Levy et al., 2008; Marshall et al., 2012). In contrast with reports of suboptimal adherence rates among athletes (Arvinen-Barrow and Walker, 2013), participants in the current study reported high levels of investment in their rehabilitation and acknowledged the critical importance of following practitioner recommendations and guidelines in achieving pre-injury levels. While objective/actual measures of adherence were not taken in the current study, interview data nonetheless reinforce the benefits of patient engagement in rehabilitation and underscore the crucial role of both patients and practitioners in facilitating adherence. Toward this end, educational programs, goal setting strategies, and effective communication techniques may all be pivotal (Heil, 1993; Evans et al., 2000), in increasing patient adherence and increasing the likelihood of an effective return to sport.

Considering the multitude of return to competition stressors, it was no surprise that athletes articulated the value of various coping skills. Although research in the area of coping skills has focused primarily on their use in mitigating injury risk (Williams and Andersen, 1998), our findings reveal the importance of coping skills throughout the rehabilitation and return-to-sport time frames. A salient finding described by participants was the relevance of both intra-individual coping resources, that is trait-like tendencies (e.g., patience, optimism) and intentional coping strategies (e.g., rational thinking, problem-solving, having other interests, planning) that athletes perceived as beneficial in enabling a return to high performance standards. Such findings reinforce previous work suggesting that elite athletes possess personality attributes that allow them to surmount difficulties such as a return to sport following injury (Johnson, 1997), and that injured athletes typically employ active coping strategies (Quinn and Fallon, 1999). From a practical standpoint the coping findings from the current study, suggest the value of encouraging athletes to use pre-existing coping resources and to employ a range of active coping strategies as they transition back into competitive sport.

Building on past research examining the role of motivation in athletes’ return to sport following injury (e.g., Podlog and Eklund, 2005), findings from the current study also emphasized the role of intrinsic motivation in energizing athletes to overcome the challenges inherent in the return to sport. These findings support a wealth of motivational research demonstrating the adaptive qualities of intrinsic motivation in facilitating persistence and promoting positive health, well-being and performance outcomes (e.g., Ryan and Deci, 2000). Further research examining the role of various forms of motivation (e.g., intrinsic, extrinsic) in predicting return-to-sport outcomes (e.g., objective performance indicators, competitive anxiety, concerns about re-injury) would be beneficial.

In discussing their experiences during their first official competition (Period 2), a range of positive and negative experiences were expressed. A key finding receiving limited attention in previous research, related to basketballers’ reflection that part of their confidence upon the return to competition was a result of their beliefs in the abilities of their rehabilitation specialists and their prescribed rehabilitation regimens. Implicit in the participants’ comments is the notion of “other-efficacy,” that is, an individuals’ beliefs regarding the skills and abilities of significant others with whom they interact (e.g., coaches; physiotherapists; Lent and Lopez, 2002). Research examining other-efficacy beliefs suggests that higher other-efficacy has beneficial implications for an individuals’ self-efficacy as well as other interpersonal outcomes such as the quality of relationships with significant others (Dunlop et al., 2011). In a rehabilitation context, only a handful of studies have examined the implications of other-efficacy beliefs for rehabilitation outcomes as well as the quality of patient-practitioner relations (Jackson et al., 2012; Greviskes et al., 2018). That professional athletes in the current study suggested the importance of beliefs in the skills and abilities of their rehabilitation practitioner, indicates the potential significance of other-efficacy beliefs for enhancing rehabilitation and return-to-competition outcomes. Further work examining relationships between other-efficacy beliefs and such outcomes could have important implications for the development of interventions aiming to increase athlete efficacy upon a return to competition following injury.

Despite the fact that athletes believed in the competence of their rehabilitation practitioners, the efficacy of their prescribed rehabilitation regimens, and the fact that a return to pre-injury levels was an ongoing process, all participants nonetheless indicated a lack of satisfaction with their initial competitive performance. In particular, participants consistently reported a dearth of confidence in their ability to effectively execute skills and the presence of dysfunctional physical sensations. This apparent paradox has not been reported previously; it suggests that although athletes may perceive themselves to be psychologically ready, the experience of actually performing and competing may provide information to the contrary. It is also worth noting that even the two athletes who reported functional skill execution, later recognized that they overestimated their initial abilities, something they attributed to the euphoria of playing again. Consistent with tenets in the Integrated Model (Wiese-Bjornstal et al., 1998) this finding suggests that cognitive appraisals may fluctuate and vary over the course of athletes return to competition. From a practical standpoint, this finding indicates there may be psychological value in athletes taking the benefit of time and “distance” before drawing conclusions about the effectiveness of their return or the quality of their overall performances.

Apprehension about one’s performance and fear of re-injury were also commonly reported emotions during the initial competition. Substantial research has shown these emotions to be salient among athletes transitioning back into competition (Carson and Polman, 2012; Brewer and Redmond, 2017) and are two of the most commonly cited impediments for not returning to pre-injury levels (Ardern et al., 2011). Collectively these findings reinforce the importance of helping athletes overcome their fears using emotional support, communication strategies and psychological interventions such as imagery or relaxation exercises (Chase et al., 2005).

In addition to negative emotions and challenging experiences athletes also reported various positive experiences and factors facilitating successful initial performance. A seldom reported finding in the return to sport literature related to athletes’ attentional focus. While two participants indicated a focus on the site of the injured area, the remaining athletes reported an external, more functional focus on performance tasks, the game plan, competitive goals, and their opponent. Researchers have demonstrated that attentional allocation is a critical predictor of performance (Wulf and Prinz, 2001; Janelle and Hatfield, 2008; Christakou et al., 2012; Di Corrado et al., 2015). Evidence also supports the contention that sport injury can induce an internal focus on the injury site and shift the performer’s attention away from essential performance cues, the latter of which may lead to decrements in performance (Taylor and Taylor, 1997; Heil, 2000; Boutcher, 2007; Gray, 2015). Indeed, Gray’s (2015) experimental work revealed that athletes recovering from injury had an internal focus of attention when executing skills which resulted in performance declines. Gray argued that “a return to a high level of performance will require the re-adoption of an external focus of attention” (p. 615). Our findings indicate that sport scientists and health professionals should evaluate the selective attention of returning athletes, and when necessary, provide attentional retraining (Gray, 2015) to help athletes remain focused on crucial performance elements.

Consistent with previous research (Podlog et al., 2015), athletes articulated the importance of realistic performance expectations in facilitating the attainment of pre-injury performance levels. Evidence suggests that many athletes have difficulties lowering their expectations and may expect an immediate return to preinjury performance levels (Taylor and Taylor, 1997; Weinberg and Gould, 2011). In our study, athletes reported that the development of realistic expectations, was largely contingent on information received from physiotherapists and athletic trainers during the rehabilitation period. Basketballers indicated that the information and guidance they received from health professionals during rehabilitation was invaluable in helping them understand and accept the gap between their preinjury capabilities and those occurring during their first official game. It may be that the combination of basketballers feeling well-informed and having realistic expectations, mitigated feelings frustration or discontent associated with perceptions of poor initial performances. From an applied standpoint, results from the current study suggest that educating athletes about potential return to sport scenarios may be instrumental in helping create realistic performance expectations, which may in turn, help reduce the intensity and/or frequency of negative emotions associated with poor initial performances. Although expectancy beliefs have been shown to predict a range of downstream outcomes (e.g., Zhu and Chen, 2013), they have received scant empirical attention in a sport injury context. Further research examining the influence of athlete expectations about an upcoming return to competition on return outcomes (e.g., competitive anxiety, confidence in skill execution, objective post-injury performance) would be beneficial.

A return to pre-injury competitive levels was also facilitated by athletes’ self-reported ability to moderate their arousal levels. Specifically, the ability to self-regulate one’s arousal was believed to be valuable in interpreting one’s activation as facilitative rather than debilitative. Indeed, it has been widely recognized that optimal arousal is required for athletes to reach peak performance (Kamata et al., 2002; Bertollo et al., 2012; di Fronso et al., 2017) and that the arousal reappraisal is consistently linked to more adaptive stress responses and superior performances (Sammy et al., 2017). Consequently, findings from the current study suggest the need to improve athletes self-monitoring and self-regulation abilities, so that they can effectively modulate arousal levels, particularly during challenging situations such as the first game back following an extended injury layoff.

Gradually, during the 6 months following their first official competition (Period 3), all athletes experienced a return to performance equal to or exceeding previous performance standards. Over time, athletes reported progressive increases in effective skill execution, and positive physical sensations, both of which were instrumental in increasing confidence in their performance capabilities. Athletes emphasized the critical importance of regaining confidence in their abilities, something which is typically diminished in the injury aftermath (Heil, 1993; Podlog and Eklund, 2006). Additional inquiry examining the use of specific confidence building strategies for returning athletes holds promise for future research.

Despite the valuable findings from the current study, a number of limitations are evident that could be addressed in future research. First, given the qualitative nature of the investigation, caution is warranted in generalizing the findings. As such, further quantitative exploration of specific findings described above, would be beneficial. Second, since the experience of professional athletes may differ significantly to that of recreational performers and exercisers (Andersen, 2001), researchers should examine the experiences of players of different competitive levels. Third, researchers should also consider the role of other personal (e.g., gender, injury type) or situational characteristics (time of injury in the season) highlighted in the Integrated model on athletes’ psychological appraisals and experiences in returning to sport following injury. Fourth, in an effort to determine the factors distinguishing effective from ineffective returners, it would be instructive to investigate the experiences of injured athletes who failed to return to pre-injury levels or quit their sporting activity. Fifth and finally, while we adopted a retrospective design in order to ascertain information from athletes who had already returned to competition, prospective designs could also be useful in enabling athletes to articulate their experiences close to their actual occurrence.

Despite these limitations, findings from the current investigation lend important insights into the factors involved in regaining and/or surpassing pre-injury performance levels. In particular, the presence of certain psychological factors such as confidence in one’s own performance capabilities and that of rehabilitation providers, coping skills, intrinsic motivation, social support (particularly emotional and information), functional physical sensations, and effective skill execution may all be vital in regaining or surpassing pre-injury performance standards. Results also suggest that returning to high levels of performance is a complex, non-linear process involving a combination of positive and negatively valenced experiences unfolding over weeks and months. Findings from the current study provide valuable insights into the personal and situational factors which can be influenced and shaped in order to facilitate a successful return to competition following injury.

## Author Contributions

CC conceived, designed and performed the experiments, analyzed the data, and wrote the manuscript. SdF reviewed drafts of the manuscript and analyzed the data. MP analyzed the data and prepared figures and tables. CR analyzed the data and reviewed drafts of the manuscript. LP conceived and designed the experiments, and wrote the manuscript. MB conceived and designed the experiments, wrote the manuscript and supervised the project.

## Conflict of Interest Statement

The authors declare that the research was conducted in the absence of any commercial or financial relationships that could be construed as a potential conflict of interest.

## Appendix

Detailed description of Interview guide.

**Table:**

Part A- Demographic Questionnaire.
Name
Age
Club
Hours of training per day
Matches per week
Injury sustained
Nature of injury (degenerative/traumatic)
Time of abscence from practice (in months)
Date of first official competition

Part B

Period I- Between the commencement of rehabilitation and the first official game

1. Can you describe how your rehabilitation and subsequent sport specific training went?
2. How did you cope with the challenges of trying to return to your pre injury level?
3. Were there any persons who you think helped you to return to pre-injury levels? How did they help you?
3. During the period between your rehabilitation and first official game, which psychological factors do you believe helped you to return to pre-injury or higher levels?
**Period II- The first official game**

1. Can you please describe for me your performance during your first official game?
2. Please tell me about the cognitive, emotional, e and physical sensations you experienced during the game?
3. During the first official game were there any psychological factors that helped you perform to or surpass your pre-injury levels? Please, tell me about it?
**Period III- 6th months later the first official game**

1. Can you please describe some of your experiences in the 6 months following your first official game back?
2. Please tell me about the cognitive, emotional, and physical sensations you experienced during the first 6 months back following your return?
3. During the 6 month time-period, were there any psychological factors that helped you perform to or surpass your pre-injury levels? Please, tell me about it?
